# Increased expression of argininosuccinate synthetase protein predicts poor prognosis in human gastric cancer

**DOI:** 10.3892/or.2014.3556

**Published:** 2014-10-20

**Authors:** YAN-SHEN SHAN, HUI-PING HSU, MING-DERG LAI, MENG-CHI YEN, YI-PEY LUO, YI-LING CHEN

**Affiliations:** 1 Department of Surgery, National Cheng Kung University Hospital, College of Medicine, National Cheng Kung University, Tainan, Taiwan, R.O.C.; 2 Department of Biochemistry and Molecular Biology, College of Medicine, National Cheng Kung University, Tainan, Taiwan, R.O.C.; 3 Department of Emergency Medicine, Kaohsiung Medical University Hospital, Kaohsiung Medical University, Kaohsiung, Taiwan, R.O.C.; 4 Department of Biotechnology, Chia Nan University of Pharmacy and Science, Tainan, Taiwan, R.O.C.; 5 Department of Senior Citizen Service Management, Chia Nan University of Pharmacy and Science, Tainan, Taiwan, R.O.C.

**Keywords:** gastric cancer, recurrence-free survival, argininosuccinate synthetase

## Abstract

Aberrant expression of argininosuccinate synthetase (ASS1, also known as ASS) has been found in cancer cells and is involved in the carcinogenesis of gastric cancer. The aim of the present study was to investigate the level of ASS expression in human gastric cancer and to determine the possible correlations between ASS expression and clinicopathological findings. Immunohistochemistry was performed on paraffin-embedded tissues to determine whether ASS was expressed in 11 of 11 specimens from patients with gastric cancer. The protein was localized primarily to the cytoplasm of cancer cells and normal epithelium. In the Oncomine cancer microarray database, expression of the *ASS* gene was significantly increased in gastric cancer tissues. To investigate the clinicopathological and prognostic roles of ASS expression, we performed western blot analysis of 35 matched specimens of gastric adenocarcinomas and normal tissue obtained from patients treated at the National Cheng Kung University Hospital. The ratio of relative ASS expression (expressed as the ASS/β-actin ratio) in tumor tissues to that in normal tissues was correlated with large tumor size (P=0.007) and with the tumor, node, metastasis (TNM) stage of the American Joint Committee on Cancer staging system (P=0.031). Patients whose cancer had increased the relative expression of ASS were positive for perineural invasion and had poor recurrence-free survival. In summary, ASS expression in gastric cancer was associated with a poor prognosis. Further study of mechanisms to silence the *ASS* gene or decrease the enzymatic activity of ASS protein has the potential to provide new treatments for patients with gastric cancer.

## Introduction

Gastric cancer is one of the most common malignancies of the gastrointestinal tract in individuals of East Asian countries, such as China, Japan and Korea (1,2). Current surgical strategies and combination chemotherapies used to treat gastric cancer have resulted in a 5-year survival rate of <24% (3,4). Liver metastasis is a common problem in metastatic gastric cancer, and fatal disease can develop, as the 5-year survival rate without surgical treatment is <10% (5). Improved understanding of the possible mechanisms of tumor progression and metastasis is needed to improve clinical outcomes.

Tumor metabolism is a key process in cancer growth and progression (6). Amino acid deprivation is one approach to treat malignant tumors (7). In the present study, we have focused on the biosynthetic enzyme argininosuccinate synthetase (ASS1, also known as ASS). Arginine is synthesized from citrulline by ASS and argininosuccinate lyase (8). ASS, a rate-limiting enzyme, is expressed primarily in the liver and kidneys (9). Decreased expression of ASS has been observed in liver, skin and pancreatic cancer (10–13). Accordingly, arginine depletion therapy with pegylated arginine deiminase (ADI) has been suggested as a potential therapy for these types of cancer (10–12). However, ASS is expressed differentially according to cell type, and therefore, the functional effects of varying levels of ASS expression in different types of tumors remain controversial. Although ASS exhibits markedly different expression phenotypes in gastric cancer, this enzyme may be involved in carcinogenesis (13–15). In the present study, we investigated the expression of ASS in human gastric cancer specimens. We hypothesized that an increased expression of ASS in gastric cancer tissues is associated with a poor outcome for patients. Downregulation of ASS expression is a potential novel approach for systemic therapy for gastric cancer.

## Materials and methods

### Patients

The present study used formalin-fixed paraffin-embedded sections of tissue samples from 11 patients with gastric adenocarcinoma. These patients were randomly selected at the National Cheng Kung University Hospital (NCKUH) between February, 2001 and May, 2006. Immunohistochemical analysis of ASS expression was performed in accordance with the following methods. Fresh specimens were collected from 35 patients with gastric adenocarcinoma who underwent radical resection at the NCKUH between August, 2003 and August, 2008. A total of 35 pairs of cancerous and matched adjacent normal gastric mucosa tissues were collected. The surgeries were performed by the same surgeon (Professor P.W. Lin). Patients with stage IV cancer who underwent palliative surgery, those who received conservative treatment, and those with gastric cancer arising from other cell types were excluded. Patients who refused to donate samples were also excluded. The demographics of the patients and the histopathological features of the tumors were collected by a retrospective review of each patient’s medical chart. The pathological staging was in accordance with the tumor, node, metastasis (TNM) staging system of the American Joint Committee on Cancer (AJCC) staging manual (16). The specimens were preserved in the Human Biobank within the Research Center of Clinical Medicine of the NCKUH.

All the patients provided written informed consent, and the study was approved by the Institutional Review Board of NCKUH (IRB number: ER-97–148).

### Immunohistochemical analysis of ASS expression

Formalin-fixed paraffin-embedded sections of gastric cancer and normal stomach were used. Sections (4 μM) were cut from each tissue array block. The sections were deparaffinized and dehydrated. After the sections were subjected to antigen retrieval in an autoclave, immunohistochemical staining was performed by incubating the sections overnight with a mouse anti-human argininosuccinate synthase antibody (BD Transduction Laboratories, San Jose, CA, USA). The sections were then incubated with an avidin-biotin complex reagent (Dako, Carpinteria, CA, USA) and the final color was developed with 3-amino-9-ethyl carbazole (AEC) (Zymed Laboratories, Inc., San Francisco, CA, USA). The sections were counterstained with hematoxylin. The immunoreactivity of the ASS protein was assessed by using a semiquantitative method and scaled according to the immunoreactive score (IRS) of Remmele and Schicketanz (17). The sections were divided into four categories on the basis of the IRS scores, which ranged from 0 to 12: negative, weak, mild, and strong. Dr H.P. Hsu assessed the lesions.

### Western blot analysis of ASS expression

Total cell lysates were prepared and analyzed by SDS-polyacrylamide gel electrophoresis as previously described (18). Immunodetection was performed by using the HRP-based SuperSignal chemiluminescent Substrate (Pierce, Rockford, IL, USA). For quantification, the bands were measured with the AlphaImager 2200 system (Alpha Innotech, San Leandro, CA, USA) and the densities of the ASS bands were normalized to those of the β-actin bands. ASS expression was quantified and described as a ratio to β-actin expression (the ASS/β-actin ratio).

### Statistical analysis

In the present study, we conducted a search of the Oncomine database (http://www.oncomine.com) (19) to systematically assess the expression level of the *ASS* gene in gastric cancer. In addition, we used the online biomarker validation tool SurvExpress (http://bioinformatica.mty.itesm.mx:8080/Biomatec/SurvivaX.jsp) (20) to identify information on the gene expression from mRNA datasets of gastric cancer. The P-value was calculated by using Pearson’s linear correlation. Data were presented as mean ± SD. All statistical analyses were carried out by using SPSS version 12.0 (SPSS Institute, Chicago, IL, USA). Univariate analysis between categorical variables was performed by using the Chi-square test. Continuous variables that did not follow the normal distribution were compared by using the non-parametric Mann-Whitney or the Kruskal-Wallis test. The association between ASS expression and recurrence-free survival of patients with gastric cancer was assessed by using the Kaplan-Meier method, and the significance was tested by using the log-rank test. P<0.05 was considered to indicate a statistically significant result.

## Results

### Transcriptome analysis of ASS expression

To determine the clinical relevance of ASS in human gastric cancer, we analyzed the expression profile of *ASS* in the Oncomine cancer microarray database (Table I). We compiled information on the expression of *ASS* in normal and cancerous gastric tissues from all of the microarray studies in the database (21–27). Eight studies demonstrated that *ASS* expression was significantly increased in several types of gastric cancer, such as gastric intestinal-type adenocarcinoma (GITA), diffuse gastric adenocarcinoma (DGA) and gastric mixed adenocarcinoma (GMA) (Table I) (21,22,24–26). In addition, three studies demonstrated that GITAs had a significantly higher expression of *ASS* than did DGAs (Table I) (22,23,27).

### Immunohistochemical analysis of ASS expression in human gastric cancer

**Figure 1:**
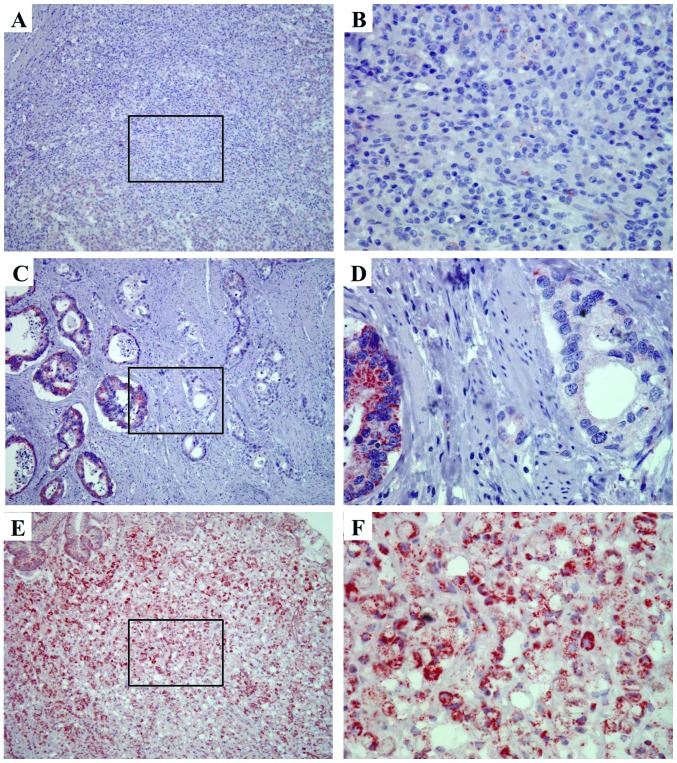
ASS expression in gastric cancer. Immunohistochemical staining to determine the distribution of ASS-positive cells (red color) was performed on paraffin-embedded specimens from AJCC stage I (A and B), stage II (C and D), and stage III (E and F) gastric cancers. (A) ASS expression was low in stage I gastric cancer (magnification, ×100). The boxed area in A is shown at a higher magnification in (B) (magnification, ×400). (C) ASS expression was moderate in stage II gastric cancer (magnification, ×100). The boxed area in C is shown at a higher magnification in (D) (magnification, ×400). (E) ASS expression was high in stage III gastric cancer (magnification, ×100). The boxed area in E is shown at a higher magnification in (F) (magnification, ×400). ASS, argininosuccinate synthetase; AJCC, American Joint Committee on Cancer.

To confirm that ASS protein was expressed in human gastric cancer, we used immunohistochemistry to determine the expression in formalin-fixed paraffin-embedded sections of whole-mount specimens. We detected ASS expression in 11 specimens (100%) from patients with gastric cancer. The protein was localized primarily to the cytoplasm of the normal and cancerous gastric epithelial cells. Examples of the expression in gastric cancer specimens are shown in Fig. 1. In a specimen from stage II cancer, we observed focal ASS expression with heterogeneity (Fig. 1C and D). Strong expression of ASS was also detected in a specimen from stage III cancer (Fig. 1E and F).

### Western blot analysis of ASS expression in clinical samples and the relationship between expression and clinicopathological parameters

**Figure 2:**
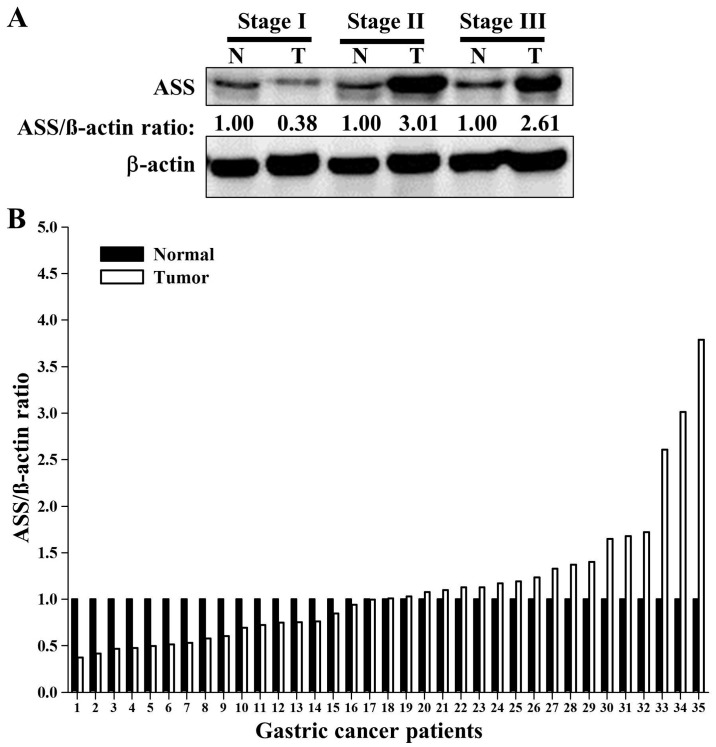
Expression of ASS protein in human gastric cancer, as detected by western blotting. (A) ASS expression was measured in specimens from gastric cancer and normal stomach. Quantitative analysis of the ratio of the expression of ASS to that of β-actin (the ASS/β-actin ratio) is shown below the immunoreactivity band for ASS. (B) Expression of ASS protein in gastric cancer and corresponding normal gastric tissues, as determined by western blotting. The expression of ASS is presented relative to that of β-actin (the ASS/β-actin ratio). The graph shows the fold change of the ASS/β-actin ratios of the 35 samples between the normal tissues (white bars) and tumor tissues (hatched bars). ASS, argininosuccinate synthetase; N, normal tissue; T, gastric cancer tissue.

We used western blots to quantify the expression of ASS in gastric cancer tissues and paired normal tissues. Quantitative analysis of ASS protein expression was achieved by normalizing the expression to that of β-actin. Fig. 2A shows the relative expression of ASS, described as the ASS/β-actin ratio, for three pairs of tissue (the results for all samples are shown in Fig. 2B). Our analysis determined that ASS expression in 19 (54%) tumor tissue samples was greater than that in the matched normal tissue samples.

**Figure 3:**
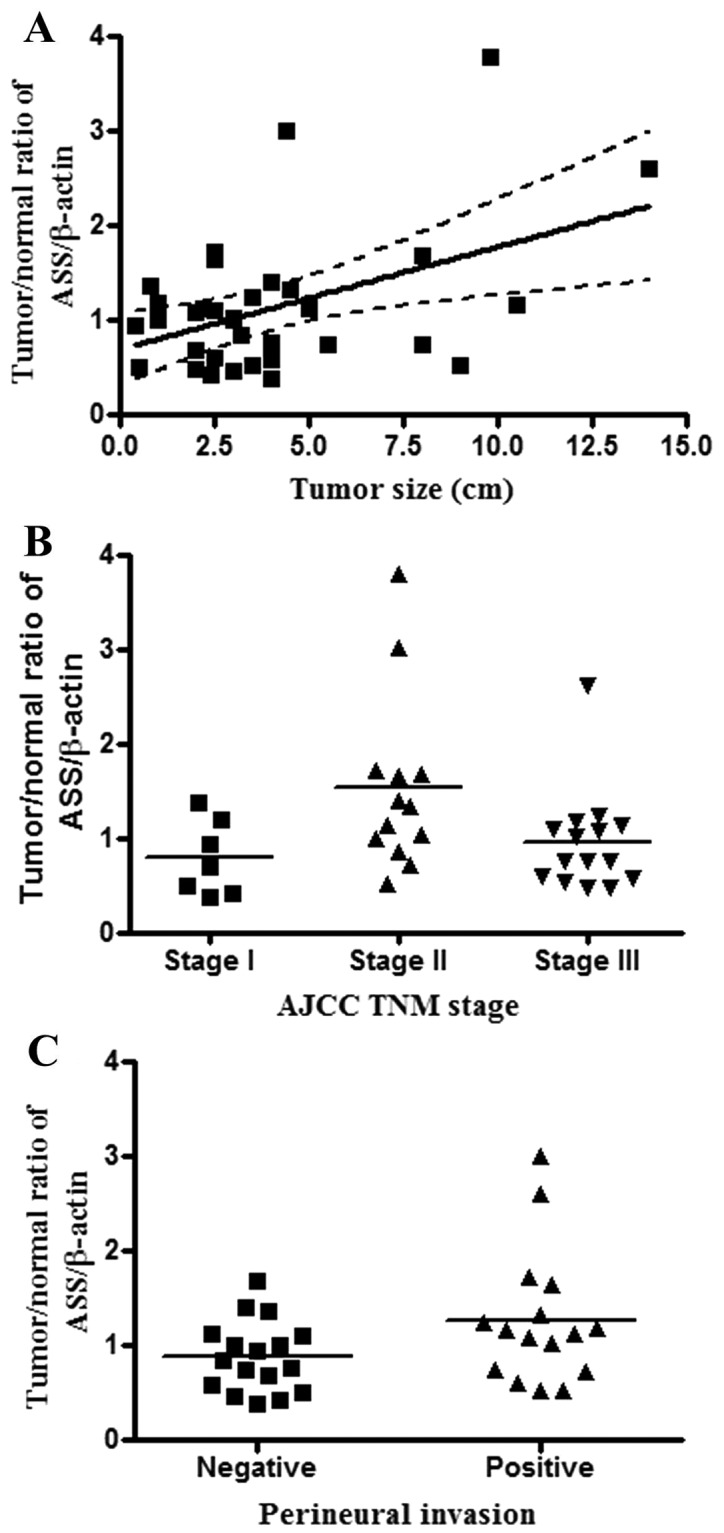
Analysis of the correlations between clinicopathological features and ASS expression in gastric cancer. The tumor/normal ratio of ASS expression (the ratio of ASS expression in specimens from gastric cancer to that in corresponding normal gastric tissues) was determined by western blotting. ASS expression is presented relative to that of β-actin (the ASS/β-actin ratio). (A) The tumor/normal ratio of ASS expression was compared with the tumor size (R^2^=0.2032, P=0.007). P-value was calculated by using Pearson’s linear correlation. (B) The tumor/normal ratio of ASS expression was compared with the AJCC TNM stage of the cancer (P=0.031). Stage II cancer was correlated with a higher tumor/normal ratio of ASS expression. (C) The tumor/normal ratio of ASS expression was compared with the perineural invasion status of the cancer (P=0.072). ASS, argininosuccinate synthetase; AJCC TNM stage, American Joint Committee on Cancer tumor, node, metastasis staging system.

We also investigated the clinicopathological characteristics of NCKUH patients with gastric cancer (Table II) and the association between these factors and ASS expression. Although the relative expression of ASS in the tumor samples was not associated with gender, age or poor predictors of histopathological factors, a higher tumor/normal ratio of relative ASS expression was associated with several clinical features (Table III; Fig. 3). First, the tumor/normal ratio of ASS expression was positively correlated with tumor size (R^2^=0.2032, P=0.007) (Fig. 3A). Second, stage II, but not stage III, cancer was correlated with a higher tumor/normal ratio of ASS expression (P=0.031) (Fig. 3B). However, one extreme value in a stage II cancer may have had substantial influence on our results (Fig. 3B). Third, while not statistically significant, the tumors that exhibited perineural invasion tended to have a higher tumor/normal ratio of ASS expression (P=0.072) (Fig. 3C). Taken together, these results suggested that ASS is involved in tumorigenesis and that it may regulate the invasive potential of gastric cancer.

### Kaplan-Meier survival analysis of patient outcomes

**Figure 4:**
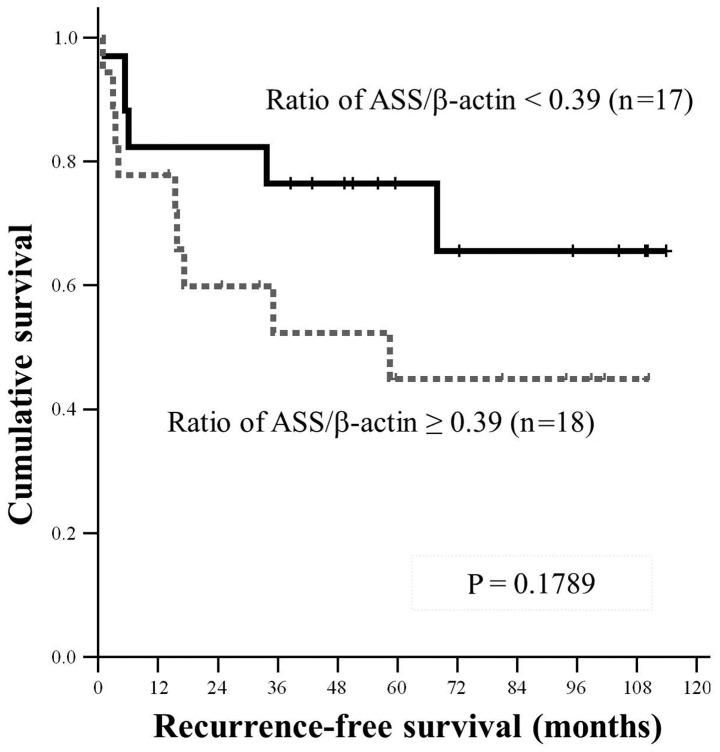
Kaplan-Meier analysis of the impact of ASS expression on recurrence-free survival in patients with gastric cancer. The expression of ASS is presented relative to that of β-actin (the ASS/β-actin ratio). The median of the ASS/β-actin ratio for all samples of gastric cancer was used as the cut-off point to create two groups: patients with a higher expression (dashed line) and those with lower expression (solid line). Patients with an increased expression of ASS had a trend toward poorer survival than patients with a lower expression (P=0.1789). ASS, argininosuccinate synthetase.

To determine whether ASS expression is correlated with outcome, we used the Kaplan-Meier method to construct survival curves. The relative expression of ASS in cancer cells and the tumor/normal ratio of ASS expression were compared with the clinical outcomes and cancer recurrence of patients with gastric cancer (Table IV). The increased expression of ASS in cancer cells and the tumor/normal ratio of ASS expression did not correlate with recurrence (Table IV). To analyze the relationship between ASS expression and recurrence, we divided the patients into 2 groups on the basis of the relative expression of ASS (the ASS/β-actin ratio). One group consisted of patients whose tumors had a relative expression less than the median value of 0.39; the other of those whose tumors had a relative expression ≥0.39. The patients in the latter group had a trend toward poor recurrence-free survival (P=0.178) (Fig. 4; Table V).

**Figure 5:**
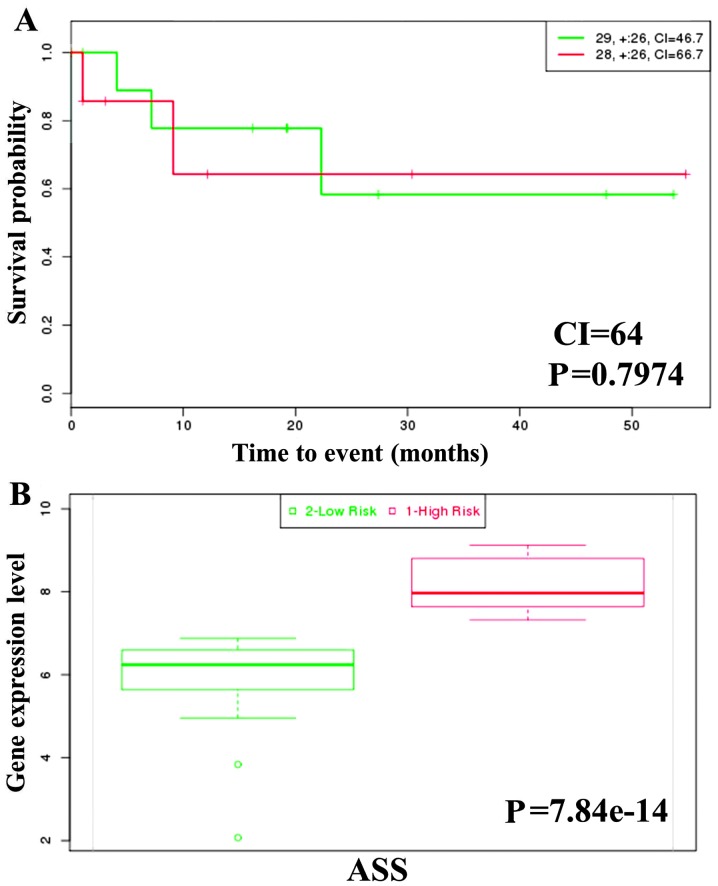
Analysis of survival and *ASS* gene expression in patients with gastric cancer. (A) Kaplan-Meier survival curves were constructed by using the SurvExpress program to analyze samples of stomach adenocarcinoma from TCGA. ‘+’ markers represent censoring samples. Low- and high-expression groups are shown in green and red, respectively. The insets in the top-right show the number of individuals, the number censored, and the CI of each risk group. (B) Box plots generated by SurvExpress show the expression levels of *ASS* and the P-value resulting from a t-test of the difference. Low- and high-risks groups are shown in green and red, respectively. ASS, argininosuccinate synthetase; CI, confidence interval; TCGA, The Cancer Genome Atlas.

To analyze the association of *ASS* expression with survival and risk, we used the online biomarker validation tool SurvExpress (20). This program derives Kaplan-Meier curves and risk groups on the basis of differences in expression. Low- and high-expression groups are shown in green and red, respectively. We determined that the expression of *ASS* in 57 samples of stomach AD from The Cancer Genome Atlas (TCGA) dataset was not correlated with survival (Fig. 5A). However, the survival risk curves derived for each group demonstrate that the expression of *ASS* was significantly increased in the high-risk group (P=7.84e-14) (Fig. 5B). In summary, the overexpression of ASS in gastric cancer is predictive of a poor prognosis. Further studies are needed to determine the manner in which ASS contributes to carcinogenesis.

## Discussion

The expression and possible roles of ASS in stomach cancer have not yet been investigated. To the best of our knowledge, the present study is the first to analyze ASS protein expression in gastric cancer. Additionally, no analyses of the correlations between clinicopathological features and prognosis of gastric cancer and ASS expression have been performed. We demonstrated that the tumor/normal ratio of ASS expression was correlated with large tumor size and advanced TNM stage. Tumors with a higher expression of ASS exhibited perineural invasion and patients with these tumors had a poorer prognosis.

In the present study, IHC analysis detected high expression of ASS in specimens from stage II and III gastric cancers. Consistent with these findings, analysis via western blotting revealed that a higher tumor/normal ratio of relative ASS expression was associated with tumor growth. Expression of ASS, as assessed by IHC, was not associated with malignant histological predictors or with poor clinical outcomes. The results of our analysis of the Oncomine database suggest that the expression of *ASS* was significantly increased in cancerous tissues (21–27). A limitation of our results is the lack of specimens from advanced, stage IV, or metastatic tumors. Despite these methodological issues, our results are consistent with the hypothesis that an elevated expression of ASS protein is associated with tumor progression.

The Kaplan-Meier method was used to evaluate the impact of ASS expression on disease-specific survival of patients. As ASS expression in normal stomach interfered with our evaluation of IHC staining, we performed western blot analysis of normal and gastric cancer tissues. Our data show that an elevated expression of ASS was significantly associated with tumor size and with a trend towards poor recurrence-free survival. The association between ASS expression and recurrence was independent of other well-known prognostic factors such as histological differentiation and World Health Organization (WHO) classification. Moreover, an increased expression of ASS in tumors was associated with perineural invasion. Metastatic gastric cancer often develops resistance to chemotherapeutic agents. In a previous study, microarray analysis revealed a significantly downregulated expression of *ASS* in patients with osteosarcoma who developed pulmonary metastases (28). Therefore, our results suggest that high levels of ASS expression correlate with the outcome of gastric cancer patients and that overexpression may affect cancer metastasis.

The prognosis of gastric cancer is poor and the 5-year survival rate is <10% (29). Inflammation is a critical component of tumor development. Persistent *Helicobacter pylori* infection has been associated with increased risk of gastric cancer (30). It has been shown that inflammation and innate immunity are associated with the development of cancer (31,32). In a recent study, it was determined that hepatic ASS is released into the circulation at very high levels in the endotoxin model of acute liver injury. This result suggests that ASS is a biomarker for inflammation (31). Increased ASS expression is associated with tumor growth at an early stage. Proinflammatory cytokines such as TNF-α and IL1-β are known to regulate ASS expression in some cancer cell lines (32,33). The multistep pathogenesis of gastric carcinoma includes superficial gastritis distal to the cardia, chronic gastritis with multifocal atrophic areas, intestinal metaplasia, dysplasia, neoplastic transformation and invasive carcinoma (32). One of these steps, the development of intestinal metaplasia, is influenced by inflammation (33). Histologically, 2 major types of gastric AD exist in the Lauren classification: intestinal and diffuse (32). Tumor cells from intestinal ADs are well differentiated and associate with intestinal metaplasia in neighboring mucosa, while those of diffuse ADs are poorly differentiated. In this study, we characterized ASS expression in gastric cancer and determined that expression was correlated with tumor progression and metastasis. Future studies should be conducted to investigate the effects of ASS silencing or overexpression in gastric cancer.

Given the results of the present study, we suggest that determining the status of ASS expression in tumor samples may be beneficial in the selection of patients for novel anticancer drug therapies. Hepatocellular carcinoma, malignant melanoma, malignant pleural mesothelioma, prostate and renal cancers are arginine auxotrophic (13). The downregulation of ASS in a tumor results in the inability to synthesize arginine for growth. Arginine depletion is a targeted therapy for various types of cancer that lack ASS expression, as dietary restriction of arginine can inhibit metastatic growth of tumor cells (34). ADI catabolizes arginine to citrulline and ammonia. A pegylated form of ADI (ADI-PEG20) has been used as an anticancer drug in clinical trials (35–37). Therefore, since gastric cancer is characterized by the overexpression of ASS, we suggest that using RNA silencing technology to downregulate *ASS* expression and ADI-PEG20 to deprive a tumor of arginine may inhibit tumor cell growth. Evaluating the level of ASS expression in gastric cancer is an important precursor to determining the type of therapy to use.

In conclusion, to the best of our knowledge, this is the first study to examine the expression of ASS protein in gastric cancer. Our results demonstrate that ASS is overexpressed in human gastric carcinoma and that expression of this enzyme correlated with tumor growth and invasion. Thus, ASS expression is a valuable prognostic indicator for gastric cancer.

## Figures and Tables

**Table I:** Analysis of Oncomine^a^ microarray studies of the expression of ASS in human gastric cancer compared with normal tissues.

Analysis type	Tissues analyzed	Samples (n)	Median expression	Fold-change	P-value	Refs.
Normal vs. cancer	Gastric tissue	19	3.408	2.158	7.57E-5	Cho *et al* (21)
	GITA	20	4.418			
	Gastric tissue	19	3.408	2.880	1.23E-5	
	DGA	31	4.777			
	Gastric tissue	19	3.408	2.477	0.004	
	GMA	10	4.779			
	Gastric tissue	15	3.692	1.523	0.035	Wang *et al* (24)
	Gastric cancer	12	3.965			
	Gastric mucosa	31	3.694	3.475	8.55E-7	D’Errico *et al* (22)
	GITA	26	5.321			
	Gastric tissue	98	−0.021	1.034	0.002	Deng *et al* (25)
	Gastric cancer	42	0.008			
	Gastric tissue	98	−0.021	1.022	0.005	
	Gastric adenocarcinoma	63	0			
	Gastric mucosa	29	−0.388	1.486	0.013	Chen *et al* (26)
	GITA	64	0.291			
Cancer vs. cancer	DGA	19	−0.409	1.750	0.003	Förster *et al* (23)
	GITA	24	0.239			
	DGA	6	3.755	2.483	0.026	D’Errico *et al* (22)
	GITA	26	5.321			
	GMA	4	5.099			
	DGA	47	5.082	1.528	0.005	Ooi *et al* (27)
	GITA	54	6.038			
	GMA	15	5.534			

a www.oncomine.org.

ASS, argininosuccinate synthetase; DGA, diffuse gastric adenocarcinoma; GITA, gastric intestinal type adenocarcinoma; GMA, gastric mixed adenocarcinoma; n, number.

**Table II:** Demographics and histopathological findings in patients with gastric cancer.

Characteristics	Participants
Total, n (%)	35 (100)
Gender, n (%)	
Male	14 (40)
Female	21 (60)
Age, mean (SD), years	64 (13)
Histological differentiation, n (%)	
Well or moderate	19 (54)
Poor	16 (46)
Lauren’s classification, n (%)	
Intestinal	20 (57)
Diffuse	11 (32)
Mixed	4 (11)
Lymph node metastasis, n (%)	
Negative	15 (43)
Positive	20 (57)
AJCC TNM stage, n (%)	
I	7 (20)
II	13 (37)
III	15 (43)
IV	0 (0)
Recurrence, n (%)	14 (40)
Clinical outcome, n (%)	
Alive	20 (57)
Death due to cancer	11 (32)
Death due to other causes	4 (11)

AJCC TNM stage, American Joint Committee on Cancer tumor, node, metastasis staging system; n, number; SD, standard deviation.

**Table III:** Correlation of ASS expression in cancer cells and the tumor/normal ratio of ASS expression with histopathological data of patients with gastric cancer.

	ASS expression^a^ in cancer cells (n=35)		Tumor/normal ratio of ASS expression^a^ (n=35)	
		
Characteristics	Median (range)	P-value	Median (range)	P-value
All patients	0.65 (0.73)^b^ 0.39 (0.02–3.74)^c^		1.13 (0.74)^b^ 1.01 (0.38–3.79)^c^	
Gender		0.459		0.699
Female	0.32 (0.11–1.36)		1.02 (0.38–2.61)	
Male	0.50 (0.02–3.74)		1.01 (0.47–3.79)	
Histological differentiation		0.791		0.274
Well or moderate	0.50 (0.02–3.74)		1.03 (0.42–3.79)	
Poor	0.34 (0.06–1.72)		0.97 (0.37–1.65)	
WHO classification		0.856		0.570
Tubular	0.50 (0.02–3.74)		1.03 (0.42–3.79)	
Mucinous	0.30		0.76	
Poorly-differentiated	0.35 (0.06–1.72)		1.08 (0.48–1.65)	
Signet-ring cell	0.32 (0.28–0.54)		0.73 (0.38–1.17)	
Lauren’s classification		0.471		0.724
Intestinal	0.48 (0.02–3.74)		1.02 (0.42–3.79)	
Diffuse	0.33 (0.06–0.70)		1.11 (0.52–1.65)	
Mixed	0.50 (0.30–1.36)		0.85 (0.38–3.01)	
Borrmann’s classification (type)		0.401		0.882
I	1.31 (0.50–2.12)		2.24 (0.69–3.79)	
II	0.32 (0.02–3.74)		1.04 (0.42–1.68)	
III	0.35 (0.08–1.72)		1.01 (0.38–3.01)	
IV	0.89 (0.06–1.36)		1.01 (0.53–2.61)	
Lymph node metastasis		0.907		0.815
Negative	0.32 (0.06–3.74)		0.94 (0.38–3.79)	
Positive	0.42 (0.02–1.72)		1.02 (0.47–3.01)	
Depth of invasion		0.747		0.396
Lamina propria (T1a)	0.32 (0.30–0.33)		0.72 (0.50–0.94)	
Submucosa (T1b)	0.50 (0.31–3.74)		0.69 (0.38–1.37)	
Muscularis propria (T2)	0.91 (0.02–1.41)		0.79 (0.47–1.72)	
Subserosa (T3)	0.40 (0.08–2.12)		1.24 (0.48–3.79)	
Serosa (T4a)	0.39 (0.21–1.72)		1.01 (0.52–1.40)	
Adjacent organ (T4b)	0.26 (0.06–1.24)		0.94 (0.53–2.61)	
Lymphovascular tumor emboli		0.970		0.924
Negative	0.32 (0.06–3.74)		0.94 (0.42–3.79)	
Positive	0.42 (0.02–1.41)		1.02 (0.38–2.61)	

a Expression of ASS protein is presented as the ratio of the expression of ASS to that of β-actin, as determined by western blotting.

b Data presented are the mean (SD).

c Data presented are the median (range).

ASS, argininosuccinate synthetase; WHO, World Health Organization; n, number; SD, standard deviation.

**Table IV:** Correlation of ASS expression in cancer cells and the tumor/normal ratio of ASS expression with clinical outcomes of patients with gastric cancer.

	ASS expression^a^ in cancer cells (n=35)		Tumor/normal ratio of ASS expression^a^ (n=35)	
		
Characteristics	Median (range)	P-value	Median (range)	P-value
All patients	0.65 (0.73)^b^ 0.39 (0.02–3.74)^c^		1.13 (0.74)^b^ 1.01 (0.38–3.79)^c^	
Clinical outcome		0.109		0.867
Alive	0.31 (0.06–3.74)		1.09 (0.39–3.79)	
Death due to cancer	0.40 (0.02–1.29)		1.01 (0.47–1.72)	
Death due to other causes	1.00 (0.50–1.72)		0.86 (0.69–1.40)	
Recurrence	0.43 (0.02–1.29)	0.933	1.02 (0.47–2.61)	0.987
No recurrence	0.32 (0.06–3.74)		1.00 (0.38–3.79)	
Liver metastasis	0.43 (0.02–1.24)	1.000	0.86 (0.47–2.61)	0.732
No liver metastasis	0.36 (0.06–3.74)		1.01 (0.38–3.79)	
Local recurrence	0.27 (0.08–0.45)	0.392	1.38 (1.03–1.72)	0.276
No local recurrence	0.37 (0.02–3.74)		0.97 (0.38–3.79)	
Peritoneal carcinomatosis	0.35 (0.02–0.51)	0.246	1.00 (0.47–1.65)	0.923
No peritoneal carcinomatosis	0.37 (0.06–3.74)		0.97 (0.38–3.79)	
Lymphoid system metastasis^d^	0.39 (0.02–0.50)	0.399	0.69 (0.47–1.72)	0.210
No lymphoid system metastasis	0.33 (0.06–3.74)		1.08 (0.38–3.79)	
Lung metastasis	0.37 (0.02–0.51)	0.569	0.53 (0.47–1.24)	0.111
No lung metastasis	0.37 (0.06–3.74)		1.02 (0.38–3.79)	
Bone metastasis	0.50 (0.35–0.51)	0.561	0.69 (0.48–1.24)	0.383
No bone metastasis	0.33 (0.02–3.74)		1.01 (0.38–3.79)	
Other metastasis^e^	0.40 (0.08–0.51)	0.674	1.24 (1.03–1.65)	0.232
No other metastasis	0.35 (0.02–3.74)		0.94 (0.38–3.79)	

a Expression of ASS protein is presented as the ratio of the expression of ASS to that of β-actin, as determined by western blotting.

b Data presented are the mean (SD).

c Data presented are the median (range).

d Including metastasis to the spleen or other intra-abdominal lymph nodes, such as the hepatoduodenal, retropancreatic, mesenteric, or para-aortic nodes.

e Including brain and colon metastases and anastomotic recurrence.

ASS, argininosuccinate synthetase; n, number; SD, standard deviation.

**Table V:** Impact of ASS expression on recurrence-free survival in patients with gastric cancer.

			Recurrence-free survival (%)					
			
Expression of ASS^a^	Patients (n)	Median survival (months)	1-year	2-year	3-year	4-year	5-year	6-year
Below median^b^	17	NA^c^	82.4	82.4	76.5	76.5	76.5	65.6
At or above median^b^	18	59	77.8	59.8	52.4	52.4	44.9	44.9
All patients	35	NA^c^	80.0	71.1	64.7	64.7	60.3	55.3

a Expression of ASS protein is shown as the ratio of the expression of ASS to that of β-actin, as determined by western blotting.

b Median expression of ASS in cancer cells was 0.39.

c Median survival has not yet been reached.

ASS, argininosuccinate synthetase; n, number; NA, not available.
